# Development and deployment of interpretable machine-learning model for predicting in-hospital mortality in elderly patients with acute kidney disease

**DOI:** 10.1080/0886022X.2022.2142139

**Published:** 2022-11-07

**Authors:** Mingxia Li, Qinghe Zhuang, Shuangping Zhao, Li Huang, Chenghuan Hu, Buyao Zhang, Qinlan Hou

**Affiliations:** a Department of Critical Care Medicine, Xiangya Hospital Central South University, Changsha, China; b School of Computer Science and Engineering, Central South University, Changsha, China; c National Clinical Research Center for Geriatric Disorders, Changsha, China; d Hunan Provincial Clinical Research Center of Intensive Care Medicine, Changsha, China

**Keywords:** Acute kidney disease, in-hospital mortality, elderly patients, machine learning, noninvasive parameters

## Abstract

**Background:**

Acute kidney injury (AKI) is more likely to develop in the elderly admitted to the intensive care unit (ICU). Acute kidney disease (AKD) affects ∼45% of patients with AKI and increases short-term mortality. However, there are no studies on the prognosis of AKD in the elderly.

**Methods:**

Data from 2666 elderly patients with AKD in the Medical Information Mart for Intensive Care IV were used for model development and 535 in the eICU Collaborative Research Database for external validation. Based on 5 machine learning algorithms, 33 noninvasive parameters were extracted as features for modeling.

**Results:**

In-hospital mortality of AKD in the elderly was 29.6% and 31.8% in development and validation cohorts, respectively. The comprehensive best-performing algorithm was the support vector machine (SVM), and a simplified online application included only 10 features employing SVM (AUC: 0.810 and 0.776 in the training and external validation cohorts, respectively) was deployed. Model interpretation by SHapley Additive exPlanation (SHAP) values revealed that the difference (AKD day – ICU day) in sequential organ failure assessment (delta SOFA), Glasgow coma scale (GCS), delta GCS, delta peripheral oxygen saturation (SpO2), and SOFA were the top five features associated with prognosis. The optimal target was determined by SHAP values from partial dependence plots.

**Conclusions:**

A web-based tool was externally validated and deployed to predict the early prognosis of AKD in the elderly based on readily available noninvasive parameters, assisting clinicians in intervening with precision and purpose to save lives to the greatest extent.

## Introduction

Acute kidney injury (AKI), a complex public health concern affecting ∼15% of patients in hospitals and 40% in intensive care units (ICUs), is associated with a high incidence of adverse events with organ involvement [1–3]. As a result of changes in kidney structure, more comorbidities, and greater susceptibility to renal injury, the morbidity and mortality of elderly patients with AKI are higher than in younger groups [4,5]. In 2017, the Acute Disease Quality Initiative (ADQI) 16 workgroup defined acute kidney disease (AKD) as recurrent renal impairment within 7–90 days of the AKI diagnosis [6]. As a novel concept in the field of kidney disease, AKD presents considerable potential for clinical research during the transitional period between AKI and chronic kidney disease (CKD) [7]. About half of patients with AKI might progress to AKD [8]. Meanwhile, the risk of death associated with AKD in ICU was approximately twice that of AKI patients [9]. Furthermore, 60% of patients in ICU were over 65 years old, reflecting the aging process of the inpatient population [10]. Being the primary focus of health care, the elderly deserve more urgent attention. Therefore, it is necessary to conduct population characterization and construct a reliable and accessible web-based tool to assess the prognosis for AKD in the elderly, which may provide a critical window for early targeted interventions.

Recently, clinical interest has gradually been drawn to research on predictive prognosis for AKD, while enthusiasm for research on AKI remains strong. There have been several studies conducted to predict AKD in patients with sepsis, post-nephrectomy, or post-cardiac surgery, with high predictive performance [11–13]. In addition, a prospective study found that urinary neutrophil gelatinase-associated lipocalin could be used as a biomarker of long-term survival of patients with AKD admitted to coronary care units [14]. Xiao et al. proposed a prognostic early warning model for hospitalized AKD patients based on the traditional logistic regression method [15]. Furthermore, Yan et al. have developed a neural network-based model to predict AKI in patients with CKD following the administration of iodinated contrast media, which has superior prediction accuracy than logical regression, but the researchers did not construct online applications for further assessment [16]. However, there are some inconveniences in operation, limited repeatability, or lack of external validation regardless of whether the model is traditional statistically or machine-learning based. Also, biomarkers have limitations similar to laboratory indicators, such as difficult acquisition, high costs, and delayed results.

Accordingly, we aimed to develop a web-based tool for predicting in-hospital mortality of AKD in the elderly based on machine learning algorithms with high accuracy and noninvasive parameters with easy access and adjustment, and further to perform external validation to demonstrate the generalization of the tool. Moreover, we employed SHAP to visualize the features in order to determine optimal thresholds for early clinical decision-making to enhance short-term outcomes.

## Methods

### Study design and cohort extraction

This was a multicenter retrospective cohort study based on large electronic medical records datasets. We included patients older than or equal to 60 years of age, according to the definition of the elderly in China. The exclusion criteria were as follows: (1) length of stay in ICU <48 h; (2) repeated admissions to ICU; (3) end-stage renal disease (ESRD); (4) no AKI or missing diagnosis data; (5) no AKD missing diagnosis data. In accordance with inclusion and exclusion criteria, information on AKD in elderly individuals was extracted from the following critical care databases: the Medical Information Mart for Intensive Care IV (MIMIC-IV, version 1.0) originated from a single-center hospital and the eICU Collaborative Research Database (eICU-CRD, version 2.0) from multi-center hospitals [17,18]. We used the MIMIC-IV cohort for model training and the eICU-CRD cohort for external validation. It has been granted access to the MIMIC-IV and eICU-CRD databases (record ID: 41817305). Our study was exempted from approval by the Institutional Review Board due to the deprivation of the data. We adhered to the Declaration of Helsinki and the Statement of the Transparent Reporting of a multivariable prediction model for Individual Prognosis Or Diagnosis [19,20].

### Noninvasive parameters collection

Noninvasive parameters with broad access and easy intervention were used as predictors of mortality in elderly patients with AKD, as follows: (1) basic demographics: age, gender, ethnicity, and body mass index (BMI); (2) severity of disease: AKI stage, Glasgow coma scale (GCS) and sequential organ failure assessment (SOFA); (3) comorbidities: sepsis, hypertension, diabetes mellitus, cerebrovascular disease, congestive heart failure (CHF), and CKD; (4) interventions: mechanical ventilation (MV), renal replacement therapy (RRT), and vasopressor use; (5) the worst value of noninvasive vital signs monitoring: heart rate, systolic blood pressure (SBP), diastolic blood pressure (DBP), mean arterial pressure (MAP), temperature, respiratory rate, and peripheral oxygen saturation (SpO2). The above-mentioned features were extracted from the day of ICU admission. In addition, regarding the GCS, SOFA, and noninvasive monitoring of vital signs, we also identified the changes between the worst value on the day of AKD diagnosis and ICU admission, that is, the fluctuation range of these indicators as modeling features, including delta GCS, delta SOFA, delta heart rate, delta SBP, delta DBP, delta MAP, delta temperature, delta respiratory rate, and delta SpO2.

### AKD definition

AKI was diagnosed and staged based on serum creatinine and urine output according to the Kidney Disease: Improving Global Outcomes guidelines for the diagnosis and management of AKI in 2012 [21]. Patients with AKD were diagnosed on the basis of the ADQI 16 workgroup consensus in 2017, which required AKI with at least stage I within 7–90 days after the initial diagnosis of AKI or before discharge [6]. In addition, the primary outcome of our study was in-hospital mortality in the elderly with AKD, which served as the predictive endpoint of our model.

Baseline creatinine was determined by the lowest normal creatinine level during hospitalization, which also applied to patients with CKD with a higher baseline creatinine level than normal. Otherwise, we used the Modification of Diet in Renal Disease Trial formula to estimate the baseline creatinine.

### Machine learning algorithms

By analyzing massive amounts of information and identifying patterns, machine learning algorithms can make intelligent predictions on newly acquired data. A total of five supervised machine learning algorithms [logistic regression model (LRM), random forest (RF), extreme gradient boosting (XGBoost), multilayer perceptron (MLP), support vector machine (SVM)] were selected to predict categorical labels. Modeling was conducted using the MIMIC-IV cohort, and external validation was carried out using the eICU-CRD cohort as a new dataset. In order to prevent overfitting and enhance generalizability, a grid search was carried out with 10-fold cross-validation to tune the parameters of the classifier, and further predictions were made to an independent dataset. On the basis of the optimal cutoff value under various algorithms, we obtained the area under the receiver operating characteristic (ROC) curve (AUC), precision-recall curves, sensitivity, specificity, positive predictive value (PPV), and negative predictive value (NPV) to evaluate classification models. Further, calibration curves were drawn for the training and validation cohorts to assess the agreement of the predicted and actual probabilities, as well as decision curves to analyze the clinical utility. The SHapley Additive Explanation (SHAP) enhanced the interpretability of machine learning models by visualizing the marginal contributions of individual features and displaying partial dependence plots to demonstrate how features contribute to and interfere with the death risk in the elderly with AKD. Finally, we selected the best-performing model from external validation for the development, and deployment of a simplified online application with the top 10 features from importance ranking.

### Statistical analysis

All statistical analyses were conducted using Python (version 3.9.7) and R (version 4.2.0) software. Statistical significance was set as a two-sided *P* value <0.05. The continuous variables conforming to a normal distribution were represented by the mean (standard deviation) and tested by the two-tailed *t*-test, while non-normally distributed variables were expressed as the median (interquartile range) and analyzed by the Mann-Whitney U-test. Furthermore, categorical variables are presented as numbers (percentages) and analyzed using the *χ*2 test. The missing values were multiply imputed by the mice package (version 3.14.0) of R software. The features selected for this study were noninvasive parameters readily available to clinicians, so there were no features with a missing ratio exceeding 20% to be removed.

## Results

### Baseline characteristics of AKD in the elderly

**Figure 1.**
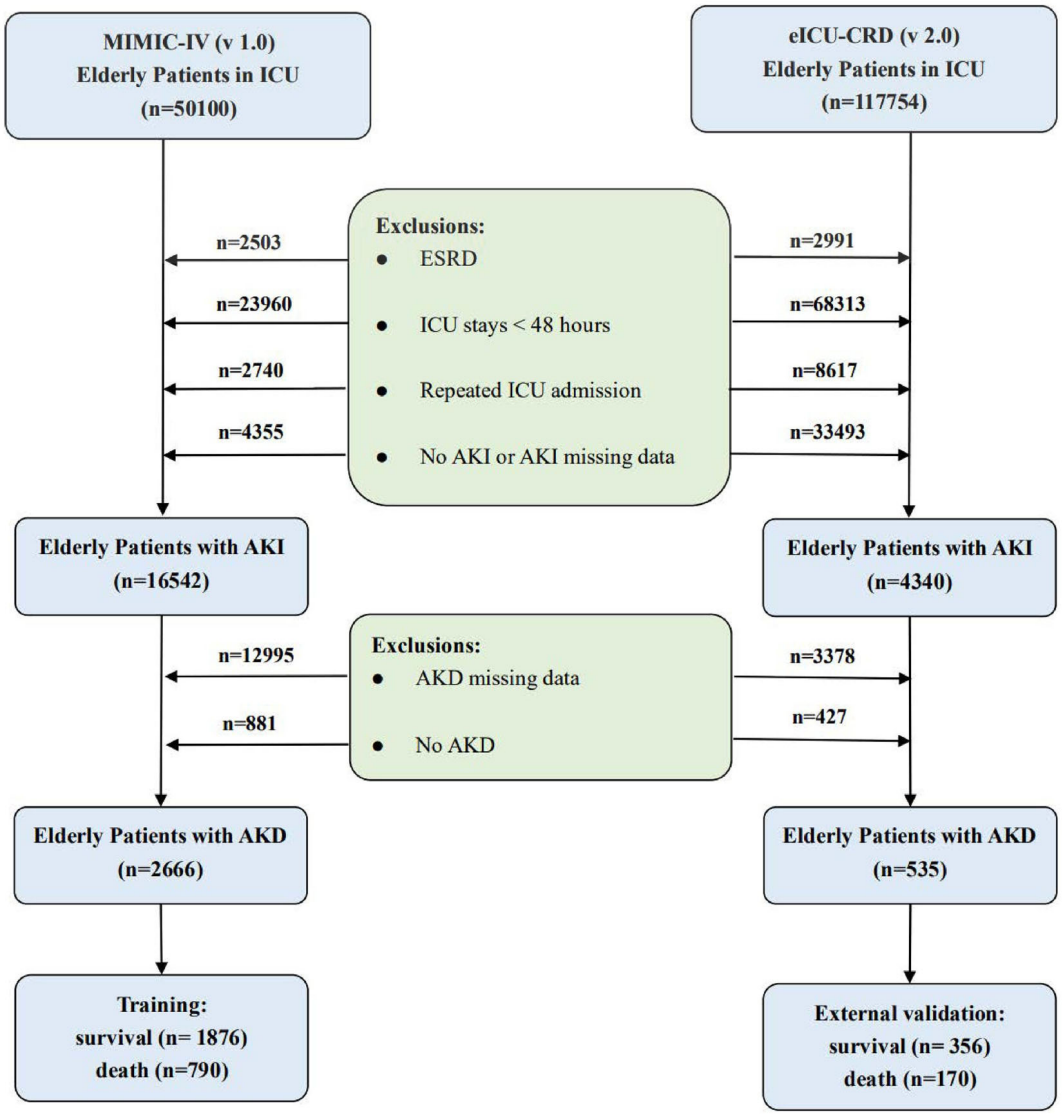
A flowchart for the selection of elderly patients with AKD. AKD: acute kidney disease; AKI: acute kidney injury; eICU-CRD: the eICU Collaborative Research Database; ESRD: end-stage renal disease; ICU: intensive care unit; MIMIC-IV: medical information mart for intensive care IV.

In this retrospective study, we finally enrolled 2666 elderly patients with AKD from the MIMIC-IV and 535 from the eICU-CRD as training and external validation datasets for the machine learning model, respectively. A detailed description of the screening process for patients is shown in Figure 1. After removing missing follow-up data from the MIMIC-IV cohort, 75.2% (2666/3547) of AKI in the elderly progressed to AKD, of whom 29.6% (790/2666) died in the hospital. Moreover, an externally validated cohort from the eICU-CRD showed an incidence of 55.6% (535/962) and in-hospital mortality of 31.8% (170/535) for elderly patients with AKD (Figure 1). According to Table 1, baseline characteristics of the two cohorts were presented, stratified by the presence or absence of in-hospital death in aged individuals with AKD. Patients with AKD who died in hospital were found to be older, have lower BMI, and be less likely to be white in terms of demographics; to have lower GCS, higher delta GCS, SOFA, and delta SOFA in terms of disease severity; to have a higher proportion of sepsis and CKD and a lower proportion of hypertension in terms of comorbidities; to be receiving more RRT and vasopressors in terms of interventional therapy; to have higher heart rate and respiratory rate and lower delta heart rate, SBP, delta SBP, DBP, delta DBP, MAP, delta MAP, temperature, delta respiratory rate, and delta SpO2 in terms of vital signs (*p* < 0.05). Furthermore, some features in the eICU-CRD cohort shared similar trends and statistical significance with the MIMIC-IV cohort, including age, BMI, sepsis, SBP, delta SBP, DBP, MAP, delta MAP, temperature, and delta SpO2 (*P* < 0.05).

**Table 1.** Baseline characteristics of elderly patients with AKD.

Variables	Training cohort			External validation cohort		
	Survival (*n* = 1876)	Death (*n* = 790)	*P*-value	Survival (*n* = 363)	Death (*n* = 172)	*P*-value
Demographic						
Age, years	72.7 [66.6, 80.1]	76.1 [68.7, 82.8]	<0.001	70.0 [64.0, 75.0]	72.0 [66.0, 79.0]	0.002
Gender, *n* (%)			0.131			0.569
Male	1087 (57.9)	432 (54.7)		200 (55.1)	100 (58.1)	
Female	789 (42.1)	358 (45.3)		163 (44.9)	72 (41.9)	
Ethnicity, *n* (%)			0.015			0.006
White	1275 (68.0)	517 (65.4)		264 (72.7)	147 (85.5)	
Black	196 (10.4)	69 (8.7)		60 (16.5)	11 (6.4)	
Asian	48 (2.6)	14 (1.8)		3 (0.8)	1 (0.6)	
Other	357 (19.0)	190 (24.1)		36 (9.9)	13 (7.6)	
BMI (kg/m^2^)	29.0 [24.8, 34.2]	27.7 [23.8, 33.0]	<0.001	29.9 [25.8, 36.0]	28.3 [24.6, 33.5]	0.018
Disease severity						
Aki stage, *n* (%)			0.583			0.141
I	1452 (77.4)	598 (75.7)		301 (82.9)	150 (87.2)	
II	389 (20.7)	178 (22.5)		58 (16.0)	18 (10.5)	
III	35 (1.9)	14 (1.8)		4 (1.1)	4 (2.3)	
GCS	10.0 [6.0, 13.0]	7.0 [3.0, 11.0]	<0.001	8.0 [3.0, 13.0]	8.0 [3.0, 13.0]	0.75
Delta GCS	5.0 [2.0, 9.0]	7.0 [4.0, 12.0]	<0.001	4.0 [0.0, 8.0]	1.0 [–2.0, 5.0]	<0.001
SOFA	8.0 [5.0, 11.0]	9.0 [7.0, 12.0]	<0.001	8.0 [5.0, 10.0]	7.0 [5.0, 10.0]	0.385
Delta SOFA	–2.0 [–4.0, 0.0]	0.0 [–3.0, 1.8]	<0.001	–1.0 [–4.0, 1.0]	1.0 [–1.2, 4.0]	<0.001
Comorbidities, *n* (%)						
Sepsis	1706 (90.9)	756 (95.7)	<0.001	107 (29.5)	71 (41.3)	0.009
Hypertension	964 (51.4)	372 (47.1)	0.047	60 (16.5)	33 (19.2)	0.525
Diabetes mellitus	757 (40.4)	291 (36.8)	0.098	53 (14.6)	35 (20.3)	0.121
Cerebrovascular disease	116 (6.2)	37 (4.7)	0.153	35 (9.6)	9 (5.2)	0.118
CHF	435 (23.2)	201 (25.4)	0.231	45 (12.4)	30 (17.4)	0.151
CKD	554 (29.5)	273 (34.6)	0.012	45 (12.4)	26 (15.1)	0.466
Interventions, *n* (%)						
MV	1849 (98.6)	780 (98.7)	0.866	65 (17.9)	44 (25.6)	0.052
RRT	311 (16.6)	228 (28.9)	<0.001	9 (2.5)	2 (1.2)	0.499
Renal toxic drugs	1811 (96.5)	767 (97.1)	0.541	54 (14.9)	46 (26.7)	0.002
Vasopressor use	1388 (74.0)	683 (86.5)	<0.001	124 (34.2)	56 (32.6)	0.789
Vital signs						
Heart rate (bpm)	85.1 [77.0, 94.3]	88.0 [78.4, 97.5]	<0.001	91.3 [80.4, 104.8]	88.9 [77.6, 98.6]	0.01
Delta heart rate (bpm)	–15.7 [–23.0, −8.6]	–17.9 [–30.1, −8.4]	<0.001	–21.8 [–34.3, −9.1]	–17.2 [–26.8, −6.4]	0.002
SBP (mmHg)	118.1 [109.5, 129.4]	112.7 [105.6, 123.2]	<0.001	116.0 (17.9)	115.0 (15.1)	0.499
Delta SBP (mmHg)	–27.7 [–38.0, −19.3]	–33.1 [–46.6, −22.5]	<0.001	–20.1 [–34.3, −3.2]	–28.6 [–43.0, −13.8]	<0.001
DBP (mmHg)	59.2 [54.2, 65.1]	57.0 [52.1, 62.4]	<0.001	62.7 [56.3, 69.8]	60.3 [55.4, 66.3]	0.003
Delta DBP (mmHg)	–16.2 [–21.8, −11.3]	–18.5 [–24.9, −12.7]	<0.001	–15.5 [–25.2, −8.8]	–17.3 [–27.3, −10.5]	0.139
MAP (mmHg)	76.4 [71.6, 82.5]	73.3 [69.4, 79.0]	<0.001	76.9 [68.7, 84.7]	74.5 [67.7, 81.4]	0.035
Delta MAP (mmHg)	–20.0 [–26.5, −13.9]	–22.4 [–32.0, −16.2]	<0.001	–16.2 [–25.4, −6.6]	–18.3 [–29.1, −9.4]	0.023
Temperature (°C)	37.0 (0.5)	36.9 (0.5)	<0.001	37.0 [36.7, 37.3]	36.7 [36.4, 37.2]	0.001
Delta Temperature (°C)	–0.6 [–0.9, –0.3]	–0.6 [–1.0, –0.3]	0.516	–0.6 [–1.1, –0.2]	–0.4 [–1.0, 0.0]	0.063
Respiratory rate (bpm)	20.3 (3.1)	21.0 (3.4)	<0.001	19.8 [17.2, 23.3]	20.0 [17.4, 24.0]	0.359
Delta Respiratory rate (bpm)	–7.1 [–9.7, –4.9]	–7.8 [–11.2, –5.3]	<0.001	–6.7 [–10.9, –3.0]	–5.1 [–10.2, –1.6]	0.038
SpO2 (%)	96.9 [95.9, 97.9]	97.0 [95.8, 97.9]	0.958	96.9 [95.2, 98.2]	96.9 [95.2, 98.7]	0.345
Delta SpO2 (%)	–5.1 [–7.3, –3.2]	–6.3 [–12.7, –3.8]	<0.001	–4.9 [–8.5, –2.0]	–7.0 [–14.9, –3.4]	<0.001

AKI: acute kidney injury; CHF: congestive heart failure; CKD: chronic kidney disease; DBP: diastolic blood pressure; GCS: Glasgow coma scale; MAP: mean artery pressure; MV: mechanical ventilation; RRT: renal replacement therapy; SBP: systolic blood pressure; SOFA: sequential organ failure assessment; SpO2: peripheral oxygen saturation.

### Performance comparison of models with machine learning algorithms

**Figure 2.**
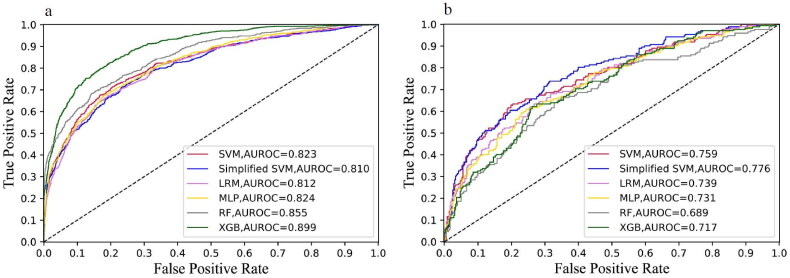
(a) The ROC curves of the SVM, Simplified SVM, LRM, MLP, RF, and XGB in the training cohort. (b) The ROC curves of the SVM, Simplified SVM, LRM, MLP, RF, and XGB in the external validation cohort. LRM: logistic regression model; MLP: multilayer perceptron; RF: random forest; SVM: support vector machine; XGBoost: extreme gradient boosting.

**Figure 3.**
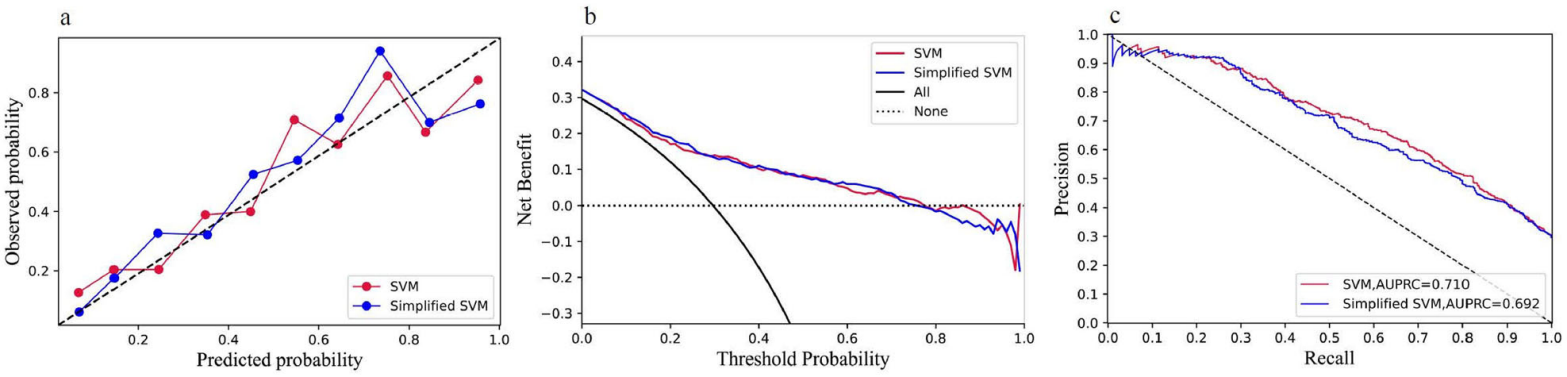
(a) Calibration curves. (b) Decision curve analysis. (c) Precision-Recall curves of the SVM and Simplified SVM in the external validation cohort. SVM: support vector machine.

As all of the variables shown in Table 1 are noninvasive and readily available in clinical practice, we incorporated all of the parameters into the development of models. The performance comparison of six machine learning models for predicting mortality of elderly patients with AKD in the training and external validation cohorts is presented in Table 2. For each algorithm in the training cohort, we performed a 10-fold cross-validation grid search to determine the optimal hyperparameters (Additional file 1), resulting in the model with the highest prediction accuracy, which was then evaluated in the testing cohort. The best prediction performance in the training cohort was achieved by XGBoost, which had an AUC of 0.899 (0.884–0.914), a sensitivity of 0.799 (0.769–0.826), a specificity of 0.830 (0.812–0.847), a PPV of 0.664 (0.633–0.694), and an NPV of 0.907 (0.893–0.921). Furthermore, in the external validation cohort, the simplified model based on SVM using the top 10 features demonstrated good discrimination for AKD in the elderly survivors, as indicated by an AUC of 0.776 (0.731–0.821), a sensitivity of 0.738 (0.666–0.802), a specificity of 0.686 (0.635–0.733), a PPV of 0.527 (0.462–0.591), and an NPV of 0.846 (0.801–0.886) (Table 2). The ROC curves for the six classification models are shown in Figure 2a,b, in which the model with the simplified model with SVM performed well in training and external validation cohorts. To illustrate the relative accuracy and clinical utility of the predictive models, we selected two models (SVM and simplified SVM) that performed better in the validation process to draw calibration curves; precision-recall curves conduct decision curve analysis (Figure 3).

**Table 2.** The predictive performance of models in the training and external validation cohorts.

Models	Training cohort					
	AUC	Cutoff	Sensitivity	Specificity	PPV	NPV
LRM	0.812 (0.793–0.832)	0.335	0.673 (0.639–0.706)	0.809 (0.790–0.826)	0.597 (0.564–0.629)	0.855 (0.837–0.871)
RF	0.855 (0.838–0.873)	0.316	0.697 (0.663–0.728)	0.845 (0.828–0.861)	0.653 (0.621–0.686)	0.868 (0.852–0.884)
XGBoost	0.899 (0.884–0.914)	0.315	0.799 (0.769–0.826)	0.830 (0.812–0.847)	0.664 (0.633–0.694)	0.907 (0.893–0.921)
MLP	0.824 (0.805–0.843)	0.356	0.675 (0.641–0.707)	0.821 (0.803–0.838)	0.613 (0.580–0.646)	0.857 (0.840–0.873)
SVM	0.823 (0.804–0.842)	0.295	0.722 (0.689–0.753)	0.787 (0.768–0.806)	0.588 (0.557–0.619)	0.870 (0.853–0.886)
Simplified SVM	0.810 (0.790–0.830)	0.271	0.713 (0.680–0.744)	0.768 (0.748–0.787)	0.564 (0.532–0.595)	0.864 (0.846–0.880)

Models	External validation cohort					
	AUC	Cutoff	Sensitivity	Specificity	PPV	NPV
LRM	0.739 (0.691–0.786)	0.293	0.634 (0.557–0.706)	0.733 (0.684–0.778)	0.529 (0.459–0.599)	0.809 (0.762–0.850)
RF	0.689 (0.639–0.739)	0.273	0.663 (0.587–0.733)	0.651(0.599–0.699)	0.473 (0.409–0.538)	0.803 (0.753–0.847)
XGBoost	0.716 (0.668–0.765)	0.246	0.622 (0.545–0.695)	0.738 (0.690–0.783)	0.530 (0.458–0.600)	0.805 (0.758–0.846)
MLP	0.731 (0.683–0.779)	0.323	0.610 (0.533–0.684)	0.755(0.707–0.798)	0.541 (0.468–0.613)	0.804 (0.757–0.844)
SVM	0.759 (0.713–0.805)	0.337	0.634 (0.557–0.706)	0.796 (0.751–0.836)	0.595 (0.521–0.667)	0.821 (0.777–0.860)
Simplified SVM	0.776 (0.731–0.821)	0.238	0.738 (0.666–0.802)	0.686 (0.635–0.733)	0.527 (0.462–0.591)	0.846 (0.801–0.886)

AUC: the area under the receiver operating characteristic curve; LRM: logistic regression model; MLP: multilayer perceptron; NPV: negative predictive value; PPV: positive predictive value; RF: random forest; SVM: support vector machine; XGBoost: extreme gradient boosting.

**Additional file 1.** The optimal hyperparameters for five models based on the ten-fold cross-validation.

Models	Hyperparameters	Settings
**LRM**	C	0.248
	max_iter	100000
	solver	liblinear
**RF**	max_depth	9
	max_features	10
	min_samples_leaf	8
	min_samples_split	4
**XGBoost**	gamma	0.2
	max_depth	4
	min_child_weight	6
	max_delta_step	0
**MLP**	kernel_initializer	uniform
	activation	hard_sigmoid
**SVM**	C	1000
	gamma	0.0001
	kernel	rbf
**Simplified SVM**	C	100
	gamma	0.001
	kernel	rbf

LRM: logistic regression model; MLP: multilayer perceptron; RF: random forest; SVM: support vector machine; XGBoost: extreme gradient boosting.

### Interpretation and visualization of SVM predictions

**Figure 4.**
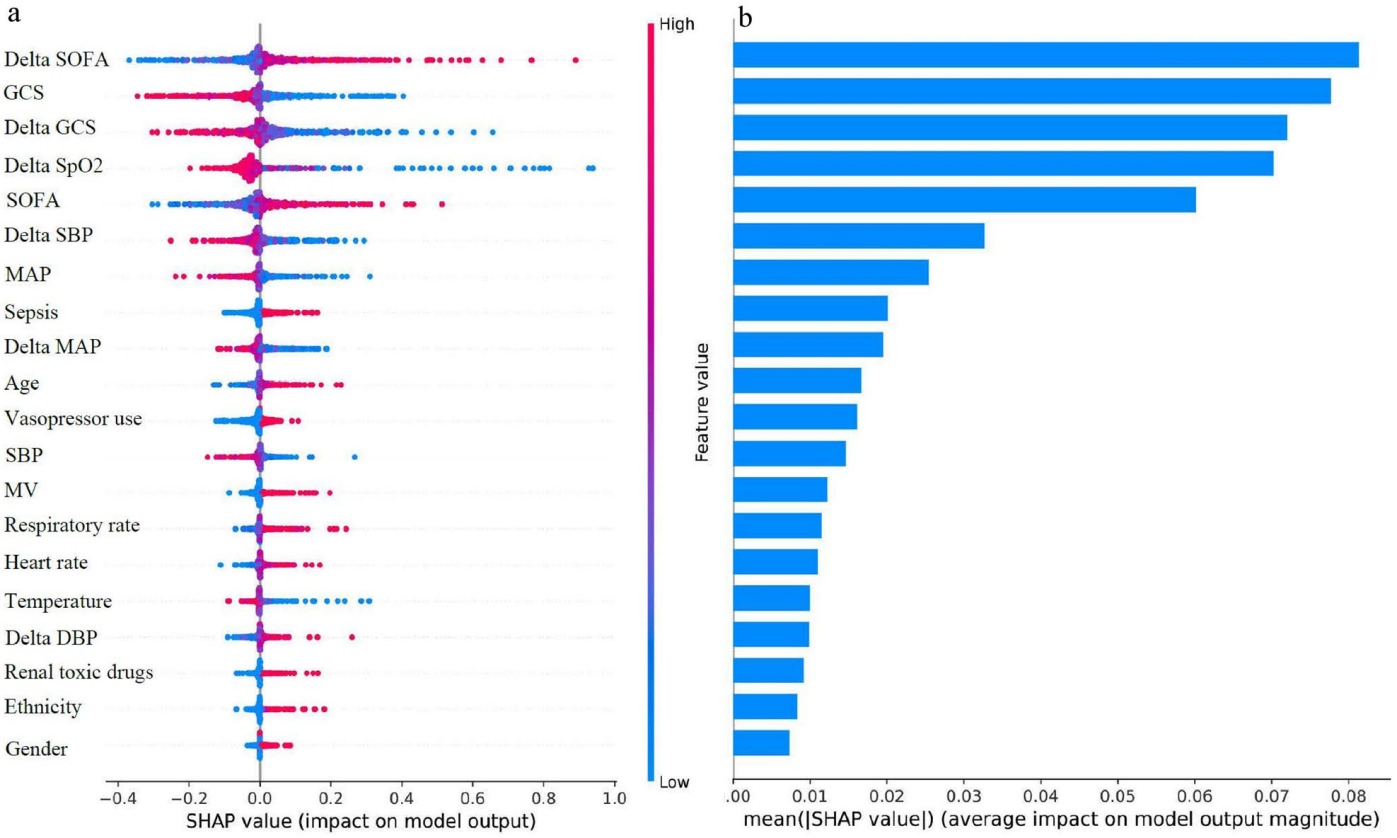
(a) The distribution of the impacts of features on the SVM model. (b) The ranking of features importance. DBP: diastolic blood pressure; GCS: Glasgow coma scale; MAP: mean artery pressure; MV: mechanical ventilation; SBP: systolic blood pressure; SpO2: peripheral oxygen saturation; SOFA: sequential organ failure assessment.

**Figure 5.**
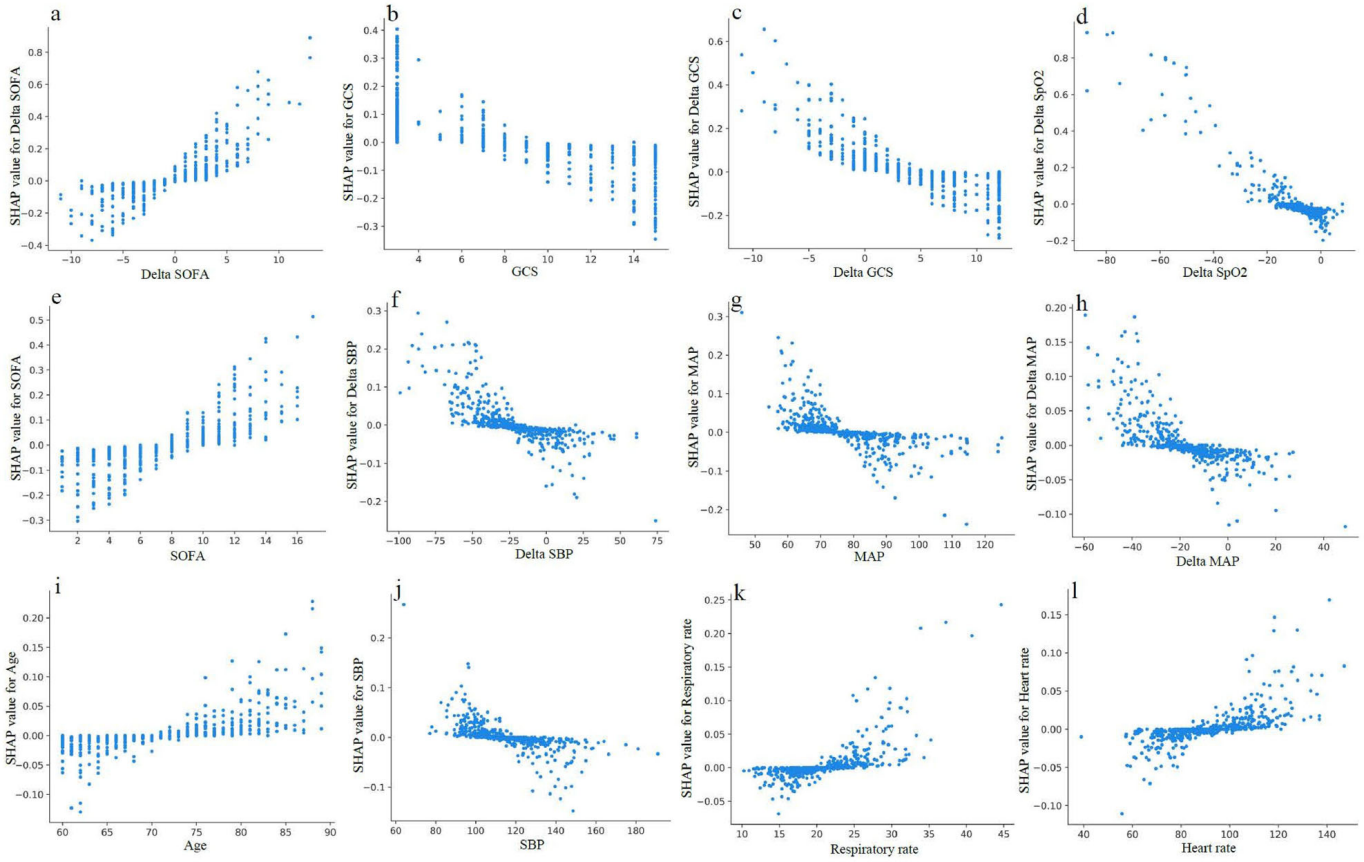
The partial dependence plots of (a) Delta SOFA, (b) GCS, (c) Delta GCS, (d) Delta SpO2, (e) SOFA, (f) Delta SBP, (g) MAP, (h) Delta MAP, (i) Age, (j) SBP, (k) Respiratory rate, and (l) Heart rate. GCS: Glasgow coma scale; MAP: mean artery pressure; SBP: systolic blood pressure; SOFA: sequential organ failure assessment; SpO2: peripheral oxygen saturation.

To better explain the clinical significance of certain variables, we utilized SHAP to visualize predictions generated by the machine learning model. As shown in Figure 4a, the risk of in-hospital mortality in elderly with AKD was positively associated with the following features: delta SOFA, SOFA, sepsis, age, vasopressor use, MV, respiratory rate, heart rate, delta DBP, renal toxic drugs, race other than white, and female. Moreover, we have drawn the ranking plot of feature importance (Figure 4b), as well as partial dependency plots for the relationship between the SHAP value and the feature value for the 12 most important continuous parameters (Figure 5). An analysis of partial dependence plots can provide a visual interpretation of the distribution of each feature and its global relationship to in-hospital mortality. Figure 5a illustrated how delta SOFA affected the risk of death. As delta SOFA gradually increased from −1, the death risk also gradually increased from 0, indicating the optimal cutoff value for delta SOFA was −1. Similar patterns can also be observed with SOFA (cutoff 7; Figure 5e), age (cutoff 75; Figure 5i), respiratory rate (cutoff 22; Figure 5k), and heart rate (cutoff 90; Figure 5l). In addition, the probability of dying in the hospital increased as GCS decreased from 10 (Figure 5b). The following features also displayed opposite trends: delta GCS (cutoff 5; Figure 5c), delta SpO2 (cutoff −10; Figure 5d), delta SBP (cutoff −25; Figure 5f), MAP (cutoff 75; Figure 5g), delta MAP (cutoff −20; Figure 5h), and SBP (cutoff 120; Figure 5j). Targeted management of vital signs based on the cutoff values shown in the partial dependence plots may contribute to controlling and minimizing the in-hospital death risk of AKD in the elderly.

### A user-friendly web-based tool for predicting in-hospital mortality in elderly patients with AKD

We finally selected the simplified SVM model using only 10 features for deployment in light of the predictive performance of various machine learning algorithms in the external validation cohort (Figure 2b), developing a readily accessible online tool for clinicians to predict in-hospital mortality of AKD in the elderly (https://forlmx.herokuapp.com/). In different clinical practice settings, once an elderly patient has been diagnosed with AKD, physicians can quickly collect and enter values for noninvasive parameters into the web application, and then click on the ‘predict’ button to obtain a prediction of in-hospital survival. The partial dependence plots in Figure 5 allowed us to treat patients at risk of death as early as possible and to control the intervenable indicators near the cutoff value, which has practical value for guiding physicians to save lives.

## Discussion

In this retrospective study using multicenter critical care data, we developed and externally validated a predictive model based primarily on noninvasive parameters for elderly patients with AKD, and then selected the best performing simplified model with SVM algorithm (AUC in the training set: 0.810 (0.790–0.830); AUC in the testing set: 0.776 (0.731–0.821)) to deploy a web-based prediction tool. According to our knowledge, this is the first prognostic study and online prediction application developed for elderly patients with AKD. More importantly, the online tool can identify patients at risk of in-hospital death on the diagnosis of AKD, allowing a larger window of time for physicians and patients to intervene.

In 2017, the ADQI 16 workgroup released an expert consensus on AKD for the first time and emphasized the need for clinical research in this area [6]. According to previous studies, ∼45% of patients with AKI progressed to AKD with in-hospital mortality of ∼26%, significantly greater than ∼12% in patients without AKD [8,9]. As a result of increased susceptibility factors to renal injury, the elderly experience higher morbidity and mortality from AKI [22]. Our study demonstrated that 29.6% and 31.8% of AKD in the elderly died in the hospital in the MIMIC-IV and eICU-CRD cohorts, respectively, which were higher than 26.1% in adults with AKD and 24.6% in sepsis with AKD from other studies [9,23]. As well, we found that in older patients with AKD, the stage of initial AKI was not associated with in-hospital mortality, similar to the multicenter study conducted by Peng et al. on AKI in the elderly [24]. Meanwhile, short- and long-term mortality was also independent of the level of AKI severity in sepsis with AKD [25].

A majority of laboratory indicators are not available on a daily basis as a result of invasive monitoring procedures and high measurement costs. Noninvasive parameters such as vital signs can be collected more conveniently and respond more sensitively to changes in the patient’s condition. Zhang et al. applied only the indicators of noninvasive monitoring to establish an outcome prediction model for general critically ill patients, and the performance was no less than that of the model incorporating laboratory indicators [26]. In addition, we found that the fluctuation ranges of certain indicators between the day of ICU admission and the diagnosis of AKD have a high predictive value and corresponding cutoff values, including delta SOFA, delta GCS, delta SpO2, delta SBP, and delta MAP. Controlling the change in indicators within the threshold range may benefit the prognosis of elderly patients with AKD. He et al. used the magnitude of SOFA change as a feature for predicting AKD in septic patients, but the delta SOFA was the difference between day 3 and day 1 without taking into account the condition at the time of AKI diagnosis [11]. Karakike et al. demonstrated that the difference in SOFA between day 7 and admission may be a reliable predictor of short-term mortality in sepsis [27]. However, no exploratory studies have been carried out on the optimal thresholds for SOFA changes at different periods of time.

GCS, SOFA, MAP, and sepsis have also been identified as critical predictors of short-term survival for elderly patients with AKD. As a measure of the level of consciousness disturbance, GCS is commonly used to assess patients with cerebrovascular disease and traumatic brain injury (TBI). A multicenter observational study showed that GCS was a critical predictor of death in hospitalization in patients with TBI [28]. Abdallah et al. reported a higher risk of 30-day mortality among adult patients with GCS of 8 or less in the emergency department [29]. In our study, we observed that GCS was inversely related to the in-hospital death risk of AKD in elderly individuals, with a critical threshold of 10 for GCS. In addition, we found that SOFA ≥7 may increase the risk of mortality for individuals with AKD, in line with previous studies indicating that SOFA was significantly associated with the prognosis of severe illnesses such as sepsis, surgery, and acute decompensated heart failure [30–32]. MAP is an essential part of hemodynamic monitoring, and the optimal threshold for treatment has been widely debated. The Surviving Sepsis Campaign Guidelines in 2021 recommended an optimal MAP target of 65 mmHg for patients with septic shock [33]. However, Maheshwari et al. demonstrated that this target setting of MAP may be prudent, since their retrospective analysis of multicenter data from 110 hospitals revealed that the in-hospital mortality of patients with sepsis increased as MAP decreased from 85 mmHg [34]. A prospective study showed that septic patients with MAP lower than 73 mmHg were more susceptible to AKI [35]. In our study, the optimal cutoff value for MAP was determined to be 75 mmHg to promote short-term survival for elderly patients with AKD. In the ICU, sepsis is the leading cause of AKI, and mortality from sepsis-associated AKI can reach 60% [36]. In a large randomized controlled trial, there was no indication that septic shock patients with AKD increased the risk of in-hospital death by 60-day and the length of ICU stay [37]. Nevertheless, our study revealed that sepsis was an independent death risk factor for people with AKD, possibly due to the broader focus on older adults.

While many studies have investigated the use of machine learning algorithms to predict prognosis, only a few have developed easy-to-use predictive tools to alert caregivers to timely interventions. In a cluster-randomized trial of multifaceted interventions in patients with AKI, an organized intervention could improve the accuracy of diagnosis and reduce the length of stay in the hospital [38]. Researchers have demonstrated the clinical value of an early warning system for AKI in identifying patients at high risk of morbidity and mortality [39]. Recently, Peng et al. have established an all-cause mortality score formula for predicting short-term survival of elderly hospitalized patients with AKI, which can be used to determine the individual death risk by adding up the scores represented by different variables [24]. However, there is currently no study on early warning of AKD in the elderly. The web-based prediction tool we developed is externally validated and can easily be deployed in any healthcare setting to guide clinicians in early intervention planning (https://forlmx.herokuapp.com/).

There are some limitations to our study. First, due to the fact that AKD was not diagnosed until 7 days after the initial event of AKI, we excluded patients who were discharged within 7 days of the diagnosis from the cohorts, since only hospitalization data was available. Second, the online prediction tool developed using multiple classifier algorithms can identify whether elderly patients with AKD are at risk of death. Therefore, the probability of death cannot be displayed in detail. Further, we are only able to predict survival when patients have been discharged from the hospital based on the available datasets, since survival data for long-term follow-up was lacking. Last, we performed external validation with the eICU-CRD cohort from the multicenter and achieved good performance. However, prospective intervention trials based on local medical record system will be necessary to determine the extent to which web prediction tools can contribute to improving the prognosis of AKD in elderly individuals as compared to clinical experience alone.

## Conclusions

In conclusion, we constructed six models for predicting in-hospital mortality of AKD in the elderly with noninvasive parameters including demographics, comorbidities, vital signs, and corresponding fluctuation differences. For generalization to different medical settings, the simplified SVM with the highest performance in the external validation cohort was selected for deployment as an online tool. As well, the predictions of the web tool combined with the optimal thresholds in the partial dependence plots have the potential to advance bundled management to improve prognosis in elderly patients with AKD.

## Data Availability

The datasets used and/or analyzed during the current study are available from the corresponding author on reasonable request.
